# Supplementary material for: Gene expression QTL mapping in stimulated iPSC-derived macrophages provides insights into common complex diseases
Source: Nat Commun. 2025 Aug 27;16:7204. doi: 10.1038/s41467-025-61670-9 (PMC12391345; doi:10.1038/s41467-025-61670-9)
Supplement: Supplementary file 1 — Supplementary Information [file 41467_2025_61670_MOESM1_ESM.pdf]

Supplementary Information

Supplementary note

iPSC culture and macrophage differentiation

iPSC culture and macrophage differentiation was carried as previously described in Alasoo, K. et al.[1] but with some minor modifications.

Feeder-dependent iPSCs were grown on irradiated CF-1 mouse embryonic fibroblast (MEF) feeder cells (AMS Biotechnology) in Advanced DMEM-F12 (Gibco) supplemented with 20% Knock-Out Serum Replacement (KSR) (Gibco), 2mM L-glutamine (Sigma), 50 IU/ml penicillin (Sigma), 50 IU/ml Streptomycin (Sigma) and 50µM β-Mercaptoethanol (Sigma M6250). The media was supplemented with 4 ng/ml recombinant human fibroblast growth factor (rhFGF) basic (R&D, 233-FB-025) to maintain pluripotency and was changed daily. MEFs were seeded on 0.1% gelatine-coated tissue-culture treated plates (Corning 6-well or 10 cm plates) 24 hours prior to passaging iPSCs at a cell density of 2 million cells/6-well or 10-cm plate in Advanced DMEM-F12 supplemented with 10% FBS (labtech), 2mM L-glutamine (Sigma), 50 IU/ml Penicillin and 50 IU/ml Streptomycin (Sigma). Prior to passaging or embryoid body formation, iPSCs were dissociated from the plates using 1:1 mixture of collagenase (1 mg/ml) and dispase (1 mg/ml) (both Gibco).

For EB formation, iPSC colonies were treated with 1:1 mixture of collagenase (1 mg/ml) and dispase (1 mg/ml) and intact colonies were transferred to low-adherence plates (Sterilin). The colonies were cultured in feeder-dependent iPSC medium without rhFGF for 3 days.

For myeloid precursor formation, EBs were harvested 3 days after formation and transferred onto gelatinised tissue-culture treated 10 cm dishes at a rate of 10 EBs per well of a 6 well plate in serum-free X-VIVO 15 (Lonza) or Stem Pro-34 SFM (Thermo Fisher), with both mediums supplemented with 2 mM GlutaMAX (Thermo Fisher), 50 IU/ml penicillin, 50 IU/ml streptomycin (Sigma), 100 ng/ml human macrophage colony stimulating factor (hM-CSF) (Peprotech) and 25 ng/ml human interleukin-3 (hIL-3) (Peprotech), changing the medium every five days. Depending on the iPSC line, myeloid precursor cells become visible as round floating cells at around 10 to 15 days in culture.

For myeloid to macrophage differentiation, myeloid progenitor cells in suspension were harvested and filtered through a 40 mm nylon filter before centrifuging at 290 g for 3 minutes before resuspending in macrophage complete media (RPMI 1640

(Thermo Fisher) supplemented with 10% heat-inactivated FBS (Thermo Fisher), 2mM GlutaMAX (Thermo Fisher) and 100 ng/ml hM-CSF (Peprotech)) at 75 cells/mL. Cells were plated at a density of 10,000 cells per well on a 96-well plate (for RNA-seq), or 25,000 cells per well of black 96-well plate (VWR) (for the macrophage purity assay) and differentiated for another 7 days.

iPSC-derived macrophage purity assay

iPSC-derived macrophages progenitor cells were seeded and differentiated as above before fixing in 50 µL of 4 % formaldehyde (Applichem, A0823.2500) at 4°C for 20 minutes. Cells were washed twice in 100 µL PBS with calcium and magnesium (Sigma, D8662) before blocking in 10% (v/v) donkey serum (AbD Serotec, C06SBZ) 0.1% Triton X-100 (Sigma, 93420) at room temperature for 1 hour. Cells were then stained with 1:200 anti-CD14 (BioLegend, 301802) and 1:800 anti-CD68 (Cell Signalling Technology 76437S) in 1% blocking solution overnight at 4C. We then washed the cells three times with PBS and stained with secondary antibodies (1:1000 donkey anti-mouse AF647 and 1:1000 donkey anti-rabbit AF488) and DAPI (10ug/mL, AppliChem A1001) at room temperature for 1 hour. Wells without primary antibody were used as negative staining controls. Cells were washed three times and imaged on a Cellomics Arrayscan (ThermoFisher), and the proportion of CD14+CD68+ cells calculated. Only cell lines with greater than 90% double stained cells were processed for RNAseq.

iPSC-derived macrophage stimulation conditions

After the 7-day differentiation to macrophages, cells were incubated for 6 or 24 hours in complete macrophage media on its own (controls) or containing the following stimuli: : 10 ng/mL recombinant human interleukin-10 (Peprotech, 200-10-2), 10 ng/mL recombinant human interferon-b (Peprotech, 300-02BC-5), 20 ng/mL recombinant human interleukin-4 (Peprotech, 200-04-5), 50 ng/mL P3C (Pam3CSK4) (Tocris, 4633/1), 20 ng/mL recombinant human interferon-γ (Peprotech, 300-02-20), 10 ng/mL lipopolysaccharides from Escherichia coli O127:B8 (Sigma Aldrich, L3129), 40 ng/mL human recombinant tumour necrosis factor alpha (Peprotech, 300-01A-10), 100 ng/mL R848 (Resiquimod) (Invivogen, tlrl-r848), or 5 ng/mL recombinant human sCD40 Ligand (Peprotech, 310-02-10). For the stimulations with HMW poly I:C (Invivogen, tlrl-pic), macrophages were transfected with poly I:C as follows: 0.15 mL of 1 mg/mL of poly I:C was mixed with 0.3 mL P3000 reagent (Lipofectamine 3000 kit) and 5 mL Opti-MEM (Thermo Fisher, 31985062). In another tube, 0.3 mL of Lipofectamine 3000 (Thermo Fisher, L3000001) and 5 mL of Opti-MEM were mixed. The diluted Lipofectamine 3000 and poly I:C tubes were mixed and incubated at room temperature for 10 minutes to allow complexes to form. 100 mL of macrophage differentiation media from above was added to the poly I:C complexes and mixed.

Media was removed from the macrophages and replaced with the diluted poly I:C complexes. Control transfections were carried out in exactly the same way but without the addition of poly I:C.

iPSC-derived macrophage low-input bulk RNA-seq preparation

At the end of the macrophage stimulation period, the media was removed and cells were lysed immediately by adding 50 µL of a 1x lysis/binding buffer (100 mM Tris-HCl pH 7.5, 0.5 M LiCl, 10 mM EDTA, 1 % w/v lithium dodecyl sulphate, and 5 mM 1,4-dithiothreitol) and mixed well. Lysed cells were stored at -80 °C until needed. Using the automated Zephyr G3 NGS Workstation (Perkin Elmer), mRNA was purified from the cell lysates in 96 well plates using the mRNA DIRECT kit (Thermo Fisher, 61012), according to the manufacturer's instructions, using 20 µL of oligo dT Dynabeads. The purified mRNA was eluted in either 7 µL of nuclease-free 10 mM Tris-HCl pH 7.5 for processing through the modified Smart-seq2 method, or 5 mL of nuclease-free water for processing through the NEBnext Ultra II Directional RNA Library kit (E7760L). For the modified Smart-seq2 method [65]), the purified mRNA was processed as follows : 2 µL of oligo $dT_{30}VN$ (Integrated DNA Technologies) and 2.34 µL of 10 mM dNTPs (Thermo Fisher, R0193) were mixed with 7 µL of the purified mRNA and heated to 72 °C for 3 minutes to denature secondary structures, before rapidly chilling on ice for 5 minutes. 5 µL of 5x SMARTScribe first-strand buffer (Clontech Takara, 639538), 0.63 µL of SUPERase inhibitor (Thermo Fisher, AM2696), 1.25 µL of 100 mM 1,4-dithiothreitol, 5 µL of betaine (Sigma, B0300-5VL), 0.15 µL of 1 M $MgCl_2$, 0.38 µL of template-switching LNA-oligo (TSO) (Qiagen) and 1.25 µL of SMARTScribe reverse transcriptase (Clontech Takara, 639538) were added to the denatured mRNA/dNTP/oligo $dT_{30}VN$ mix. Following a brief vortex mix, reverse transcription was performed at 42 °C for 90 minutes, followed by 10 cycles of 50 °C for 2 minutes, then 42 °C for 2 minutes. The reaction was stopped by incubating at 70 °C for 15 minutes. The first-strand cDNA was purified using 0.8 volumes of Ampure XP beads (Beckman Coulter, BCAG0006) to 1 volume of the reverse transcription reaction volume, according to the manufacturer's instructions, but leaving the eluted cDNA in 12 µL of 10 mM Tris-HCl pH7.5 with the beads in solution. This was done to maximise the amount of cDNA carried forward to the subsequent cDNA amplification reaction. The cDNA was amplified by adding 0.5 µL of 10 µM ISPCR primer (Integrated DNA Technologies) and 12.5 µL of 2x KAPA HiFi polymerase (Kapa Biosystems, KK2601) to the 12 µL of cDNA and mixed before heating at 98 °C for 3 minutes, followed by 11 cycles of 98 °C for 20 seconds, 67 °C for 15 seconds and 72 °C for 6 minutes, followed by a final extension at 72 °C 5 minutes. The amplified double-stranded cDNA was purified as before, but this time the Ampure XP beads were removed from the 20 µL eluate. Amplified double-stranded cDNA was quantified with a Quant-iT™ dsDNA high sensitivity assay kit (Thermo Fisher, Q33120) in black v-bottom 96-well plates (Greiner Bio-One, 651209) on a FLUOstar Omega (BMG Labtech), according manufacturers' instructions. For cDNA tagmentation, 4 ng of cDNA was diluted with 10 mM Tris-HCl pH 7.5 to a volume of 9.5 µL. 5 µL of a 3x tagmentation buffer (99 mM Tris acetate,

198 mM potassium acetate, 30 mM magnesium acetate and 48 % v/v N,N-dimethylformamide) and 0.5 µL of TDE1 (Illumina, 20034197) were added, mixed and incubated at 55 °C for 5 minutes. The tagmentation reaction was stopped by the addition of 2.5 µL of a tagmentation stop buffer (220 mM EDTA and 1.1 % w/v sodium dodecyl sulphate) and mixed before incubating at room temperature for 10 minutes. The tagmented cDNA was diluted with 10 mM Tris-HCl pH 7.5 to a final volume of 50 µL, before purifying with a 2:1 ratio of Ampure XP beads to sample volume, eluting the tagmented cDNA in 7 µL of 10 mM Tris-HCl pH 7.5. Tagmented cDNA samples were then amplified and sample-indexed by PCR as follows: 7 µL of tagmented cDNA was added to 2.5 µL of i5 index adapter and 2.5 µL of i7 index adapter from the Nextera® XT index kit v2 set A (Illumina, 15052163), 0.25 µL of 50 µM PC1 primer, 0.25 µL of 50 µM PC2 primer and 12.5 µL of 2x KAPA HiFi polymerase, before mixing and incubating at 72 °C for 3 minutes, 98 °C for 30 seconds, followed by 9 cycles at 98 °C for 15 seconds, 62 °C for 30 seconds and 72 °C for 30 seconds, followed by a final extension at 72 °C for 3 minutes. Individual libraries were purified, excess primers removed by performing 0.8:1 ratio of Ampure XP beads to PCR volume, eluting the finished library in 20 µL of 10 mM Tris-HCl pH 7.5.mRNA processed through the NEBnext Ultra II Directional library kit was done so according to the manufacturer's instructions with 17 cycles of PCR.

All libraries were quantified with a Quant-iT™ dsDNA high sensitivity assay kit, as mentioned above, before combining 96 libraries per pool in equimolar amounts. Library pools were assessed for fragment length and quantity on a Bioanalyser using a High Sensitivity DNA kit (Agilent Technologies, 5067-4626), according to the manufacturer's instructions. Each 96-library pool was sequenced over 8 lanes of a HiSeq SBS v4, collecting 75 bp paired-end reads.

References

1. Alasoo, K. *et al*. Shared genetic effects on chromatin and gene expression indicate a role for enhancer priming in immune response. *Nat. Genet.* **50**, 424–431 (2018).

Supplementary Figures

Supplementary Figure 1

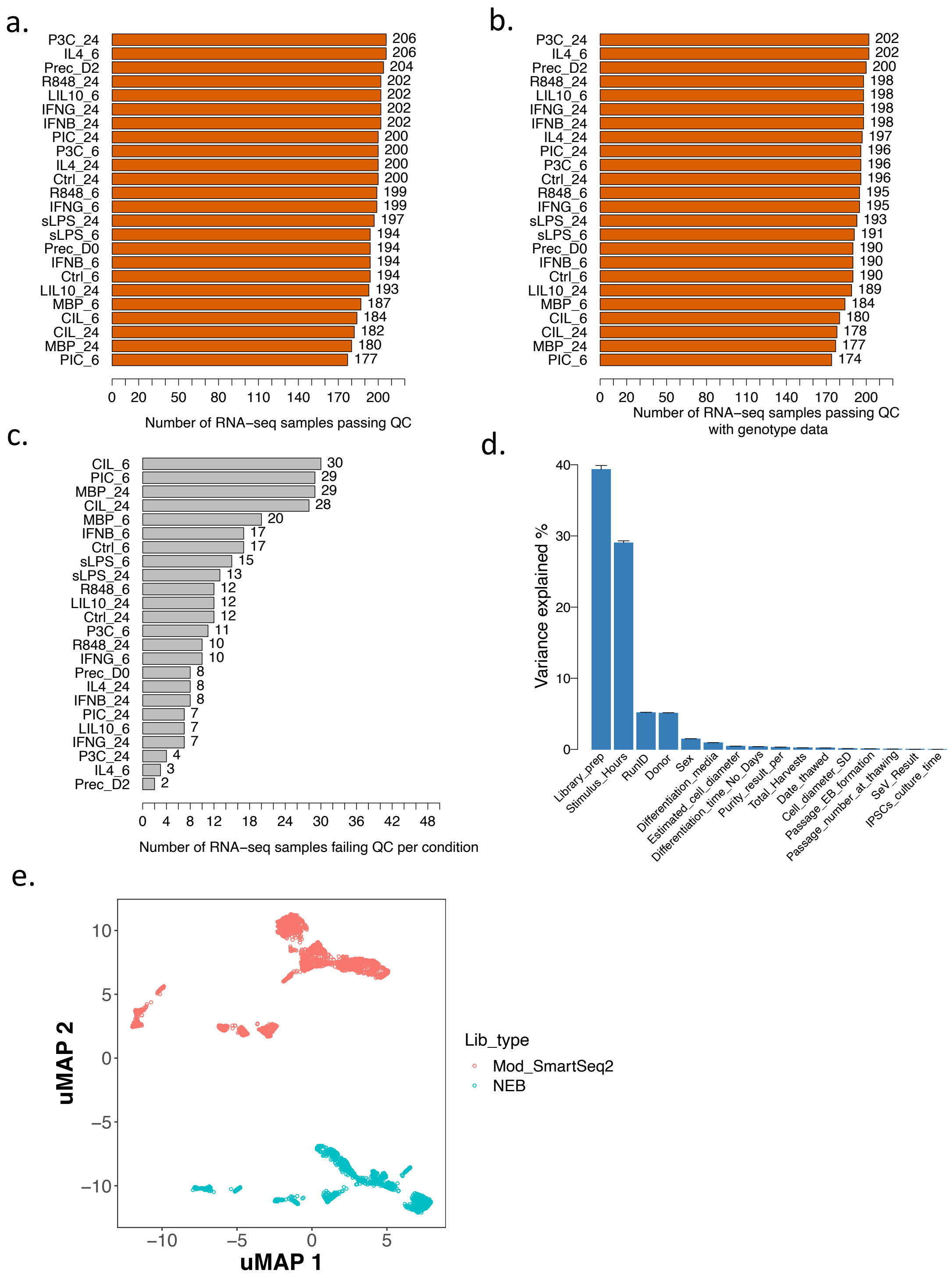

Supplementary figure 1: Comprehensive assessment of RNA-seq data quality.
a. Number of RNA-seq samples per condition that passed the quality control metric (Methods). **b.** Number of RNA-seq samples that had matching genotype data per condition. **c.** Number of

RNA-seq samples per condition failing quality control metrics (Methods) **d.** Variance deconvolution analysis taking into account multiple technical and biological covariates that could influence gene expression. **e.** UMAP representation of gene expression data without regressing out the Library Preparation method. The use of two different library preparation protocols, modified SmartSeq2 and NEBnext Ultra II Directional RNA Library kit (NEB), caused a strong batch effect that is clearly captured in the UMAP 2 and is included as a covariate in all downstream analyses.

Supplementary Figure 2

a. b.

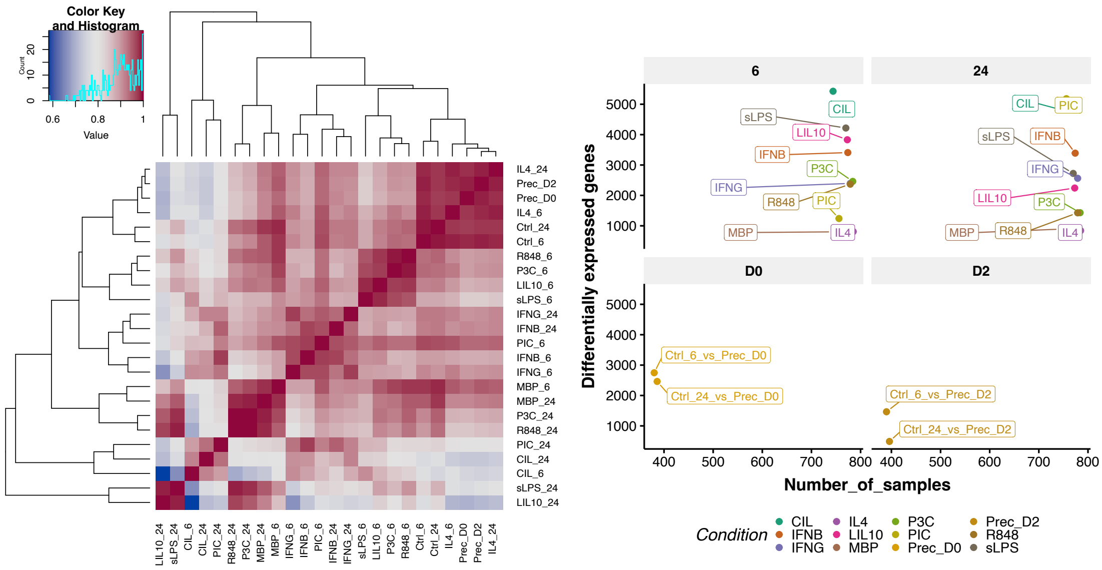

Supplementary figure 2: Impact of stimulation on gene expression: differential expression analysis and gene expression correlation clustering.

a. Heatmap and clustering of correlation of expression of quantified genes (mean TPM values per gene across all individuals) for a given condition. **b.** Number of differentially expressed genes per condition after 6 and 24 hours of stimulation compared to naïve conditions. The differentially expressed genes were defined at false discovery rate (FDR) = 5% and fold change ≥2.

Supplementary Figure 3

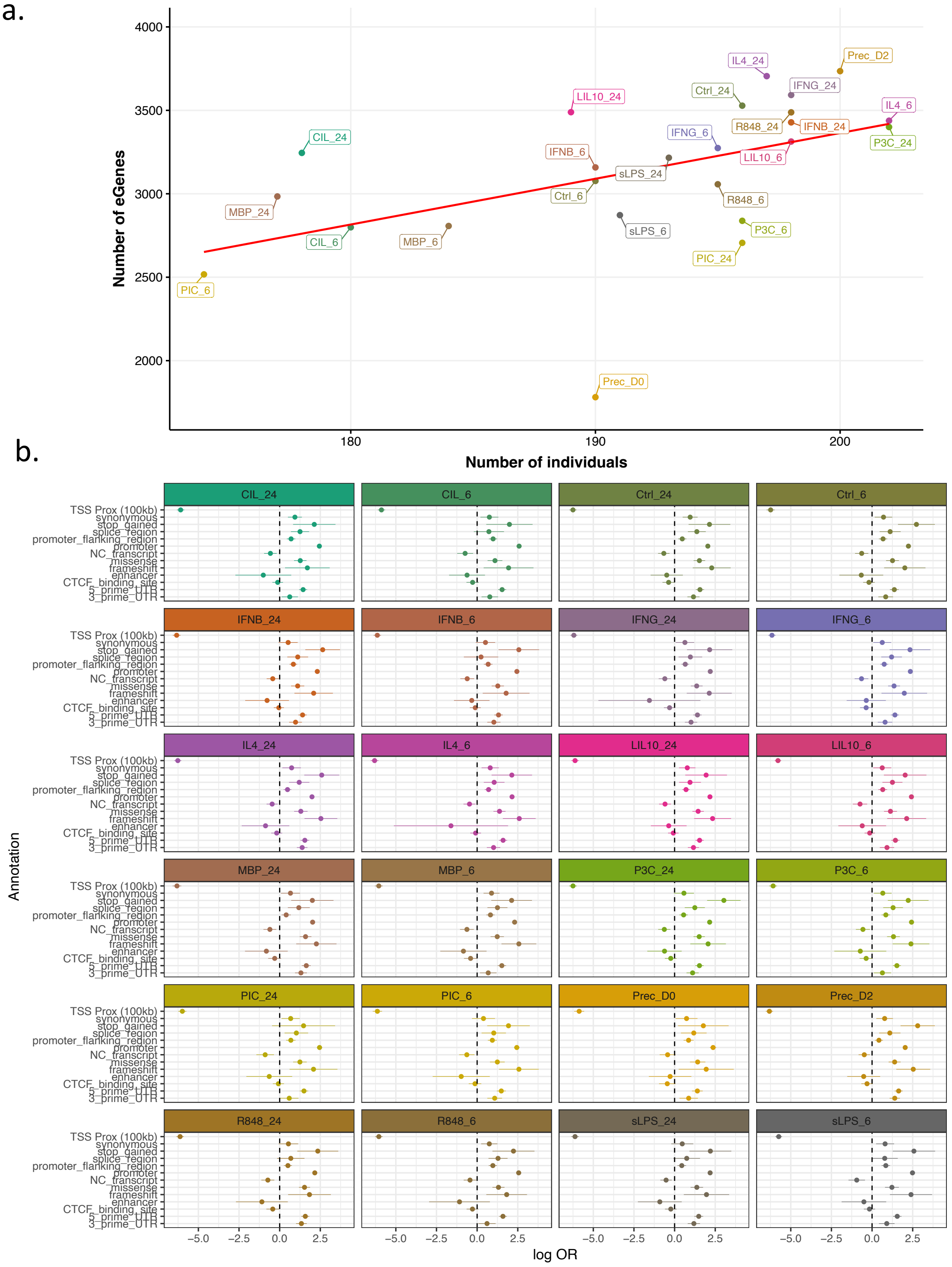

Supplementary figure 3: eQTL discovery and its functional implications.

a. Number of genes with a cis-eQTL (eGenes) per condition as a function of the sample size.

b. Enrichment analysis of eSNPs for each condition in functional annotations. Enrichment is shown as logOR. The depletion of TSS proximity means that every 100Kb further away from the TSS the number of eSNPs is decreasing.

Supplementary Figure 4

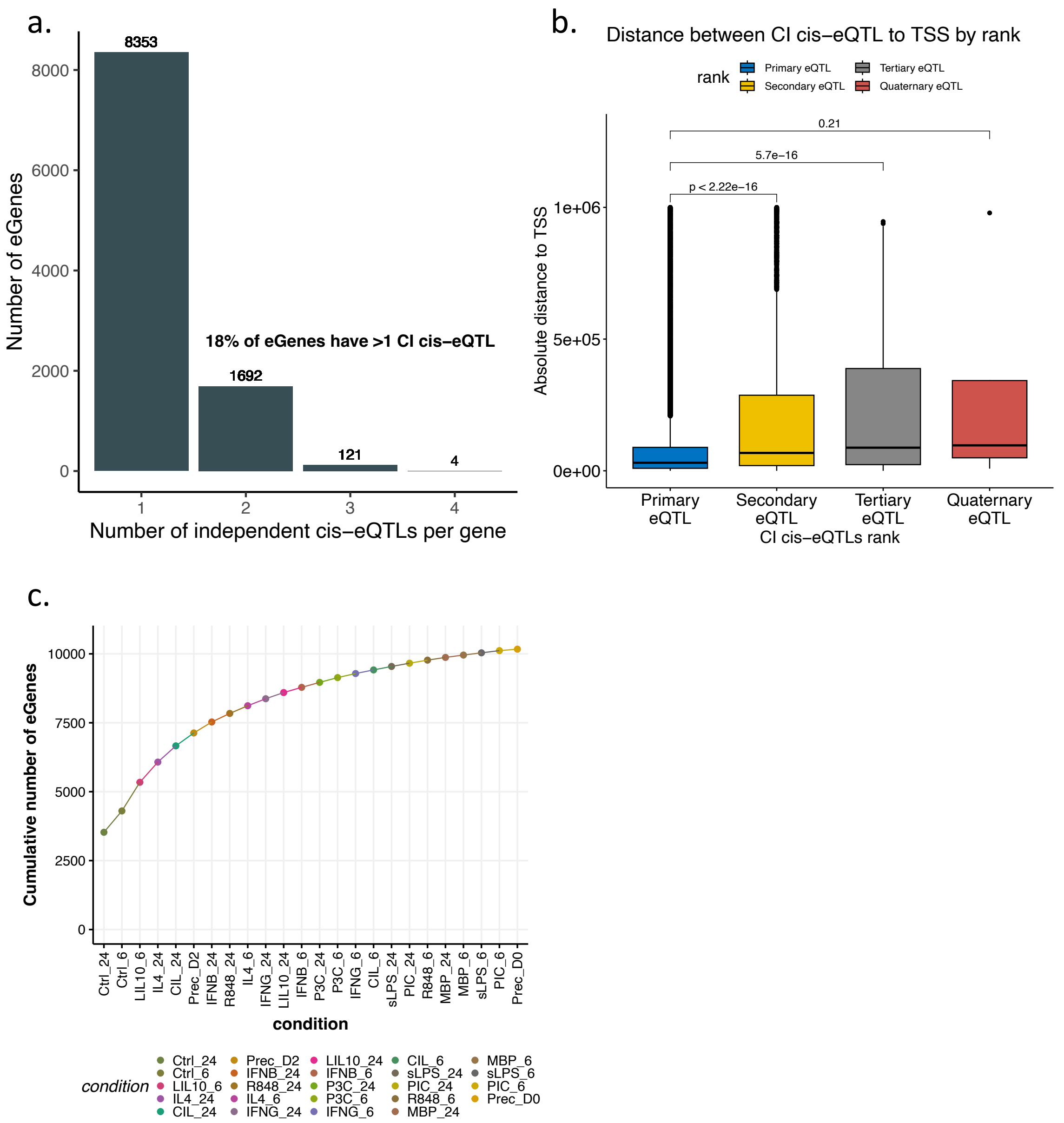

Supplementary figure 4:Total number of eGenes across conditions and conditional eQTL mapping.

a. Number of eGenes with multiple independent cis-eQTLs based on conditional analysis (Methods). b. Distribution of distances of conditionally independent eQTLs from the transcription start site (TSS) of their corresponding eGenes. c. Cumulative number of eGenes seeded in naïve conditions (Ctrl_24,Ctrl_6), with stimulated conditions shown in increasing order.

Supplementary Figure 5

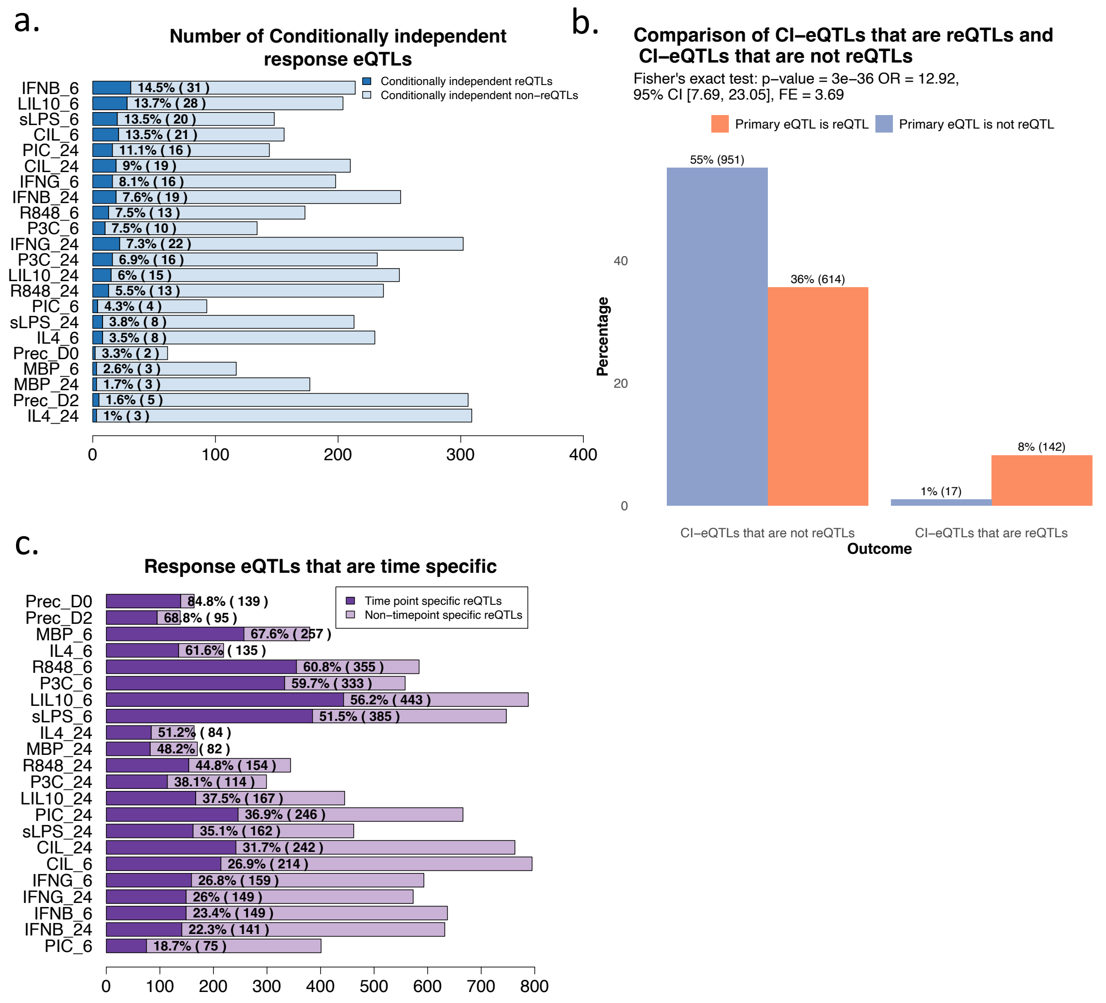

Supplementary figure 5: Landscape of conditionally Independent reQTLs and reQTLs across different time points.

a. Number and proportions of conditionally independent reQTLs per stimulation condition ordered from highest to lowest proportion. **b.** Enrichment of conditionally independent eQTLs (CI-eQTLs) that are reQTLs compared to those that are not reQTLs. Out of a total of 159 genes with a conditionally independent CI-reQTL, 142 genes (89%) also had a primary reQTL. This represents an almost 4-fold enrichment, with Fisher's exact test p-value $=3^{-36}$ and an odds ratio of 12.92 (95% confidence interval 7.7 - 23.05). **c.** Number and proportions of time point specific response QTLs ordered from highest to lowest proportion.

Supplementary Figure 6

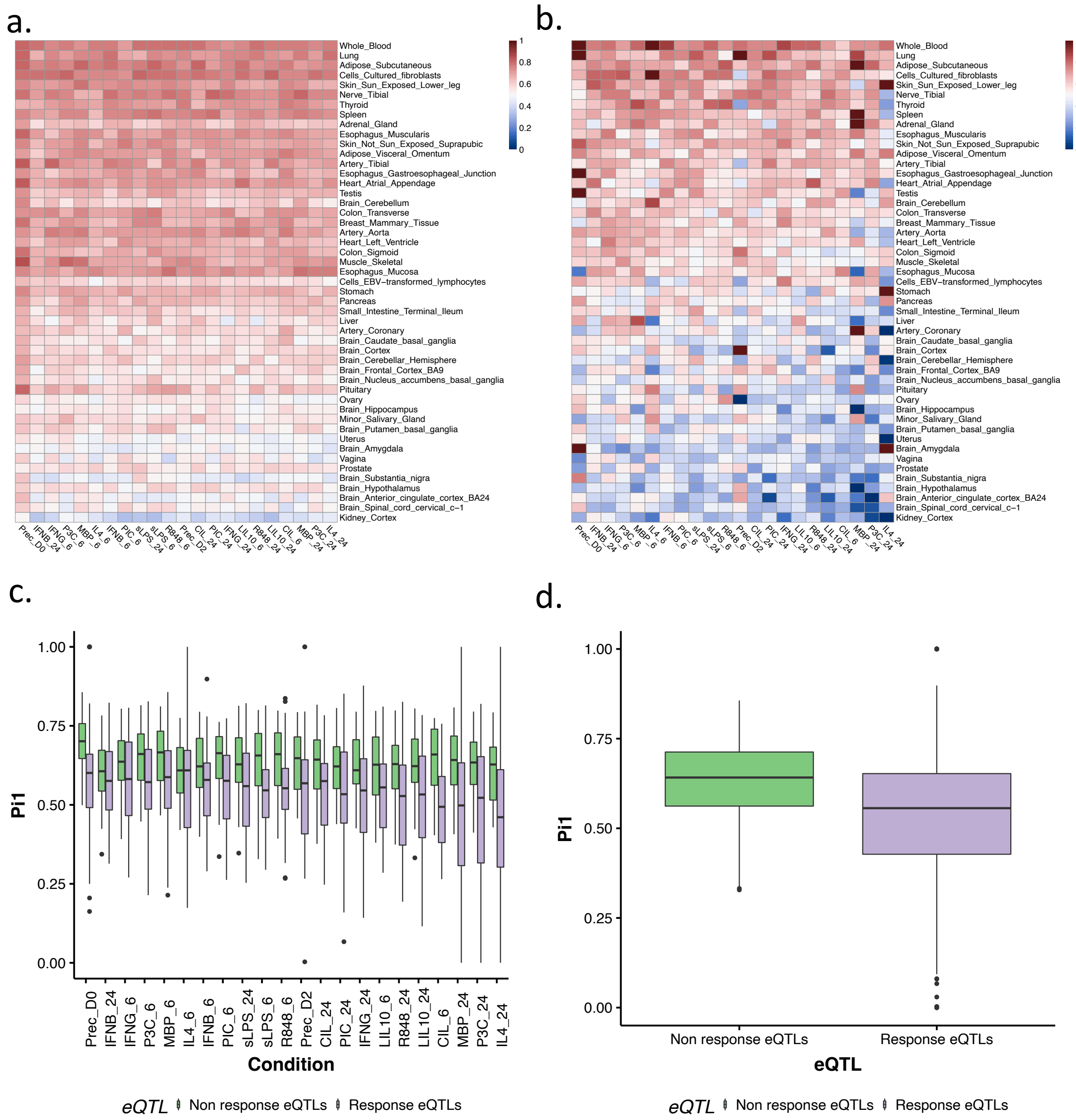

Supplementary figure 6. Detailed information on the replication rate of non-response and response QTLs-(reQTLs).

a. Heatmap displaying π1 values for non response eQTLs and GTEx tissues. The π1 values are arranged in order from tissues with the highest replication rate to those with the lowest. Higher π1 values indicate a higher replication rate. **b.** Heatmap following the same ordering as in panel (a), but for response eQTLs in all GTEx tissues.**c.** Distribution of π1 values for non response (green) and reQTLs (purple) per condition. **d.**. Distribution of the mean π1 values (across all GTEx tissues) for non response and reQTLs across all conditions. The analysis shows that reQTLs (purple) have a significantly lower replication rate based on π1 compared to non response eQTLs (green), with a Wilcoxon P-value = 4.9×10^{-47}.

Supplementary Figure 7

a.

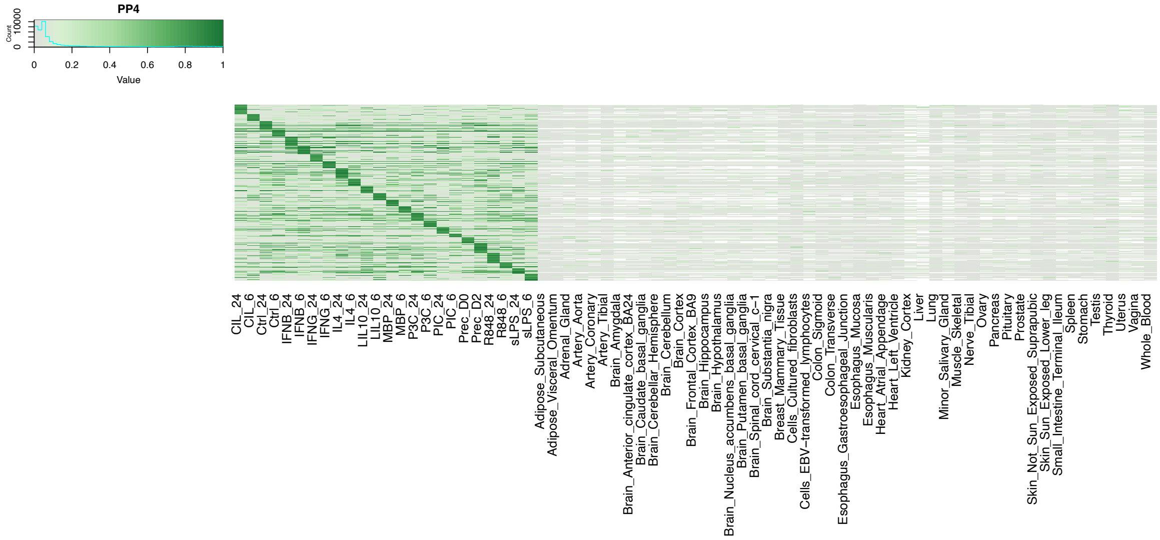

Supplementary figure 7: Colocalization evidence of eGenes with GWAS traits across GTEx tissues and MacroMap conditions.

a. Heatmap showing the posterior probability of colocalization (PP4) for the 988 eGenes with higher colocalization evidence with one or more GWAS traits in MacroMap (PP4 >0.75) compared to GTEx (PP4 <0.5) across all GTEx tissues and conditions.

Supplementary Figure 8

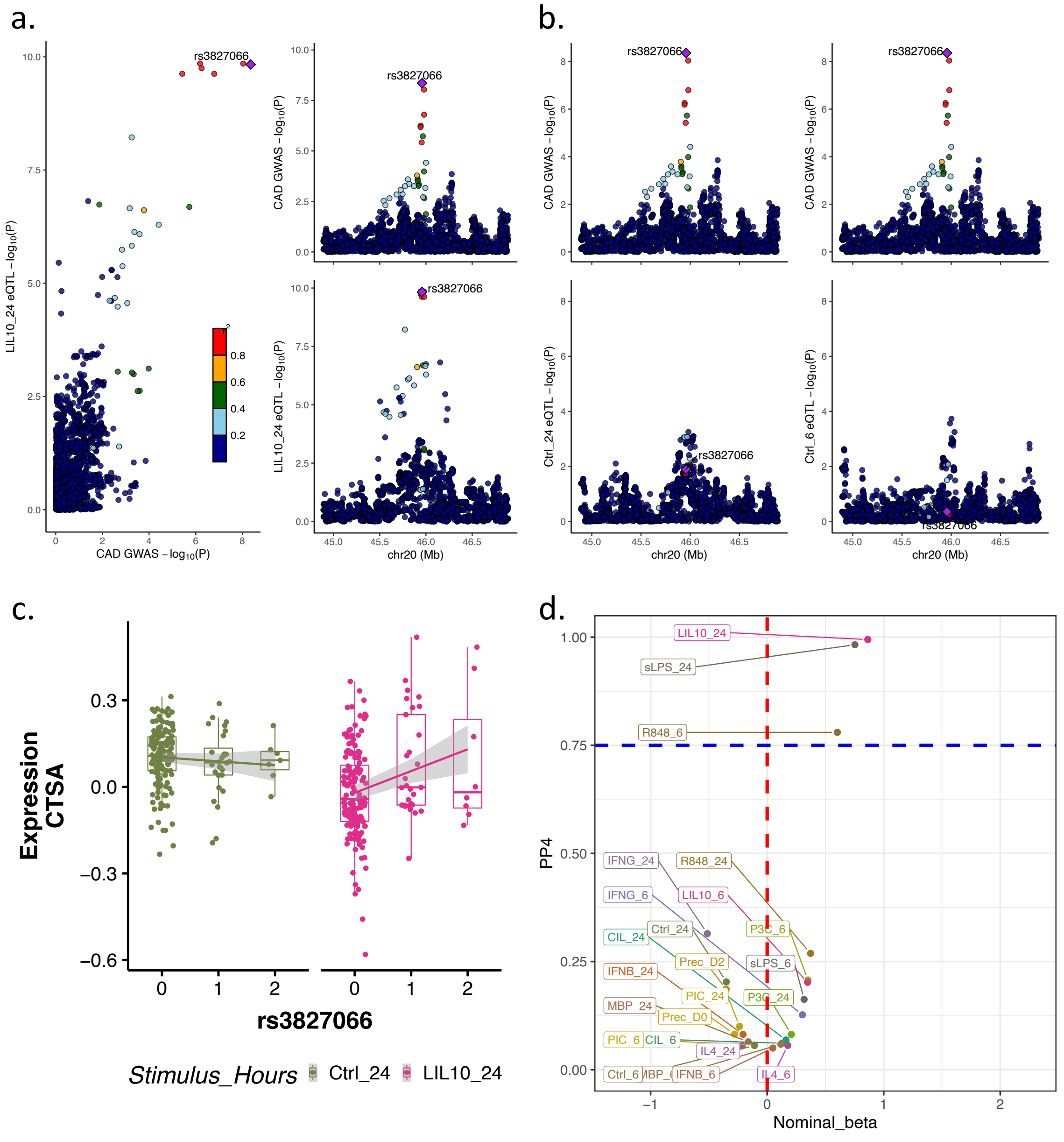

Supplementary figure 8: Colocalization between CAD GWAS hit and CTSA reQTL following LIL10 stimulation at 24h time point.

a. Scatterplot and Manhattan plots of an apparent colocalization between CAD GWAS hit rs3827066 and CTSA reQTL after stimulation with LIL10 at the 24h time point, with the purple diamond representing the lead GWAS variant. **b.** Manhattan plots of the GWAS hit rs3827066 and both naive conditions (Ctrl at 24h/6h time point) where there is no detectable eQTL effect. **c.** rs3827066 is a reQTL following stimulation with LIL10_24 with higher expression for individuals carrying the alternative genotype (0=CC,1=CT,2=TT). **d.** Scatterplot which depicts the nominal betas (x-axis) (CTSA eQTL analysis in all conditions) and posterior probability of colocalization (PP4,y-axis) for the GWAS CAD variant (rs3827066) and CTSA eQTL summary statistics. Conditions with higher nominal betas show higher colocalization evidence (PP4).

Supplementary Figure 9

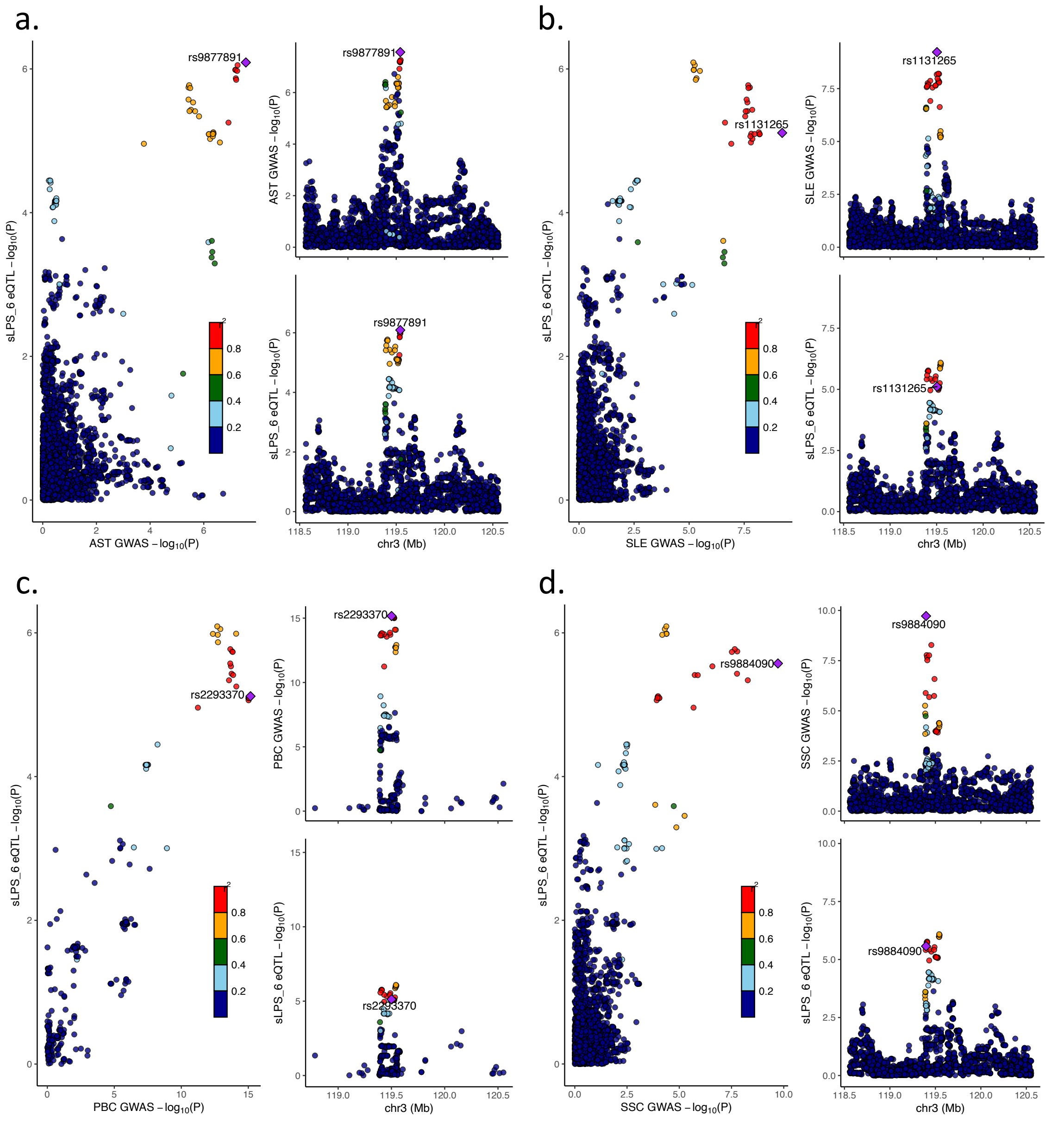

Supplementary figure 9: Colocalization of CD80 eQTL with GWAS hits in autoimmune diseases.

a-d. Scatterplot and Manhattan plots demonstrate the colocalization between CD80 eQTL and four different GWAS studies in autoimmune diseases, namely **(a)** AST/Asthma (rs9877891,PP4=0.93), **(b)** SLE/Systemic Lupus Erythematosus (rs1131265,PP4=0.88) **(c)** PBC/Primary biliary_cirrhosis (rs2293370,PP4=0.91) and **(d)** SSC/Systemic Scleroderma (rs9884090,PP4=0.93) all following stimulation with sLPS at the 6h time point.

Supplementary Figure 10

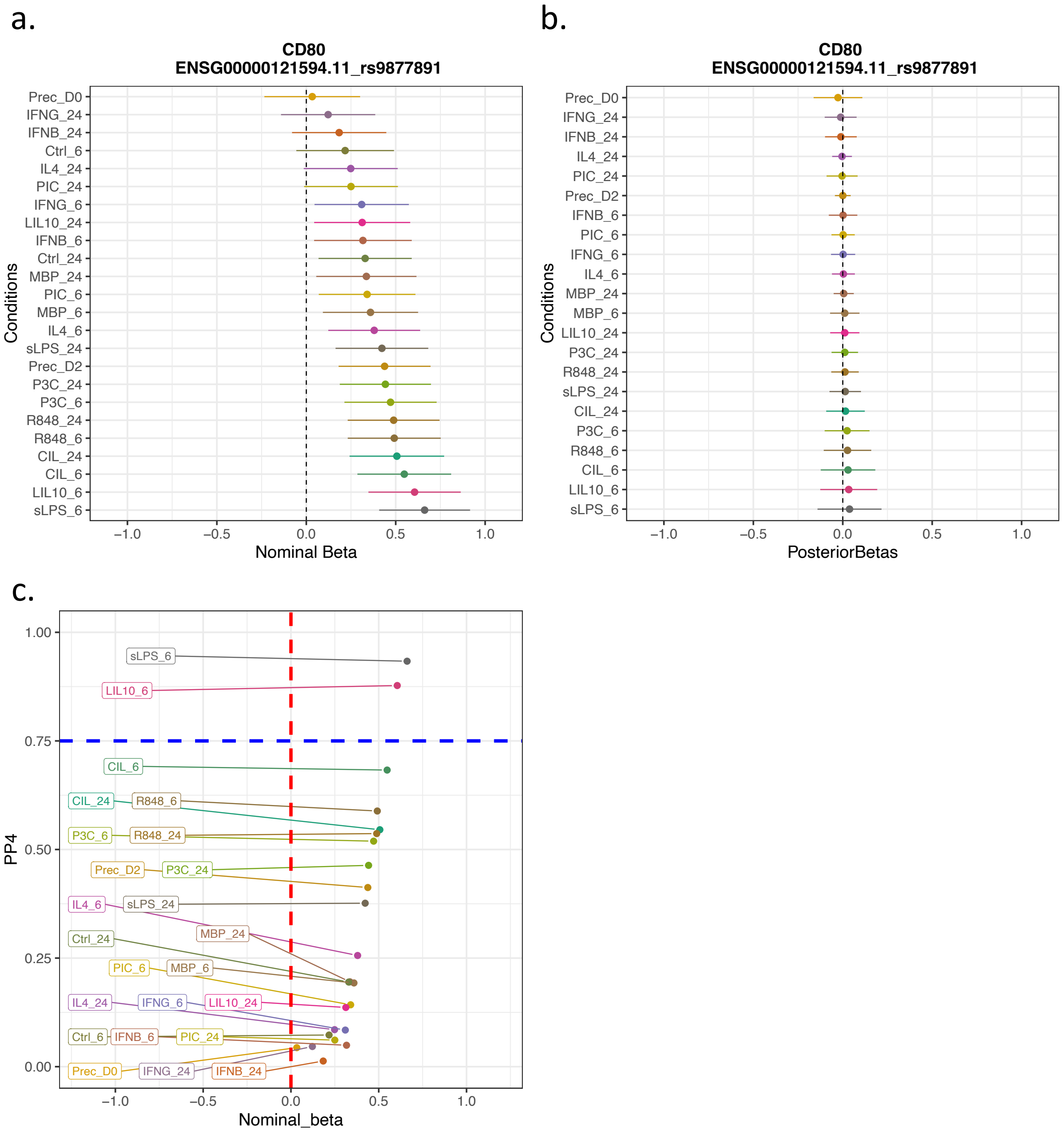

Supplementary figure 10: Exploring the potential of rs9877891 as a reQTL using condition-by-condition eQTL analysis and colocalization evidence.

a. Metaplot of nominal betas and variances for all conditions in the condition-by-condition eQTL analysis. Specifically, it displays the results for the lead eQTL variant (rs9877891) in the sLPS_6 condition for CD80, which is also the lead GWAS variant for AST/Asthma (as shown in Supplementary Figure 9a). **b**.Metaplot which shows that mash analysis did not detect rs9877891 as a reQTL. Mash estimates the extent to which the eQTL effect size in each stimulated condition deviates from that of the baseline condition (Ctrl_24) and shrinks aggressively all effects towards zero. As a result, the effect size estimates for rs9877891 are very small. **c.** Scatterplot of the nominal betas (x-axis) (condition-by- condition eQTL analysis, all conditions) for the lead eQTL variant (rs9877891, in the sLPS_6 condition) and posterior probability of colocalization (PP4,y-axis) with AST GWAS hit. Conditions with slightly higher betas (sLPS_6, LIL10_6) compared to other conditions show strong colocalization evidence

suggesting that the variant might be a true reQTL which remains undetected based on mash analysis.
