# Supplementary material for: Gene expression QTL mapping in stimulated iPSC-derived macrophages provides insights into common complex diseases
Source: Nat Commun. 2025 Aug 27;16:7204. doi: 10.1038/s41467-025-61670-9 (PMC12391345; doi:10.1038/s41467-025-61670-9)
Supplement: Supplementary file 2 — Description of Additional Supplementary Files [file 41467_2025_61670_MOESM2_ESM.pdf]

Supplementary Data Legends

Supplementary Data 1:
Description and details of the stimulation panel, including stock concentrations, working concentrations, and dilution steps for various conditions used in the experimental setup.

Supplementary Data 2:
Results of differential expression analysis comparing naive against all other conditions at 6 hours. The analysis was performed using DESeq2, applying Wald tests for differential expression. The statistical tests were two-sided, and results include the log2 fold change (log2FC), Wald statistic (stat), and P-values for each gene. P-values were adjusted for multiple comparisons using the Benjamini-Hochberg method, and adjusted P-values (padj) are reported. Genes with an absolute log2 fold change ($|\log2FC|$) ≥ 1 and adjusted P-values (padj) < 0.05 were considered statistically significant.

Supplementary Data 3:
Results of differential expression analysis comparing naive against all other conditions at 24 hours. The analysis was performed using DESeq2, applying Wald tests for differential expression. The statistical tests were two-sided, and results include the log2 fold change (log2FC), Wald statistic (stat), and P-values for each gene. P-values were adjusted for multiple comparisons using the Benjamini-Hochberg method, and adjusted P-values (padj) are reported. Genes with an absolute log2 fold change ($|\log2FC|$) ≥ 1 and adjusted P-values (padj) < 0.05 were considered statistically significant.

Supplementary Data 4:
Results of differential gene expression analysis for time point-specific effects, comparing naive and stimulated conditions at 6 and 24 hours. The analysis was performed using DESeq2 applying Wald tests for time point-specific effects (interaction term). The statistical tests were two-sided, and results include the log2 fold change (log2FC), Wald statistic (stat), and P-values for each gene. P-values were adjusted for multiple comparisons using the Benjamini-Hochberg method, and adjusted P-values (padj) are reported. Genes with an absolute log2 fold change ($|\log2FC|$) ≥ 1 and adjusted P-values (padj) < 0.05 were considered statistically significant.

Supplementary Data 5:
Gene Ontology (GO) enrichment analysis results. Differentially expressed genes (FDR < 5%) between naive and stimulated conditions at 6 hours were analysed. Significant GO terms (FDR < 0.05) are listed with their gene ratios, P-values, adjusted P-values, and associated genes.

Supplementary Data 6:
Gene Ontology (GO) enrichment analysis results. Differentially expressed genes (FDR < 5%) between naive and stimulated conditions at 24 hours were analysed. Significant GO terms (FDR < 0.05) are listed with their gene ratios, P-values, adjusted P-values, and associated genes.

Supplementary Data 7:
Reactome pathway enrichment analysis results. Differentially expressed genes (FDR < 5%) between naive and stimulated conditions at 6 hours were analysed. Significant pathways (FDR < 0.05) are presented with their gene ratios, P-values, adjusted P-values, and associated genes.

Supplementary Data 8:
Reactome pathway enrichment analysis results. Differentially expressed genes (FDR < 5%) between naive and stimulated conditions at 24 hours were analysed. Significant pathways (FDR < 0.05) are presented with their gene ratios, P-values, adjusted P-values, and associated genes.

Supplementary Data 9:
Description of GWAS studies used for colocalization

Supplementary Data 10:
Results of colocalization analysis for eQTLs and GWAS summary statistics, reporting regions with colocalization posterior probability (PP4) > 0.75. The file includes the condition name, GWAS trait, gene (HGNC), chromosome, and transcription start site (TSS). For each region, the lead eQTL and GWAS variants are detailed, including their genomic positions, P-values, and colocalization posterior probabilities (PP0–PP4).

Supplementary Data 11:
Metadata for samples that passed quality control, including information on sample identifiers, experimental conditions, sequencing details, cell culture parameters, differentiation processes, and final harvest characteristics.

Supplementary Data 12:
Description of supplementary tables and information about the datasets of the study.
