# Supplementary material for: Gene expression QTL mapping in stimulated iPSC-derived macrophages provides insights into common complex diseases
Source: Nat Commun. 2025 Aug 27;16:7204. doi: 10.1038/s41467-025-61670-9 (PMC12391345; doi:10.1038/s41467-025-61670-9)
Supplement: Supplementary file 15 — Reporting Summary [file 41467_2025_61670_MOESM15_ESM.pdf]

nature portfolio

Corresponding author(s): Carl A. Anderson

Last updated by author(s): 23/02/2025

Reporting Summary

Nature Portfolio wishes to improve the reproducibility of the work that we publish. This form provides structure for consistency and transparency in reporting. For further information on Nature Portfolio policies, see our Editorial Policies and the Editorial Policy Checklist.

Statistics

For all statistical analyses, confirm that the following items are present in the figure legend, table legend, main text, or Methods section.

n/a	Confirmed	
☐	☒	The exact sample size (n) for each experimental group/condition, given as a discrete number and unit of measurement
☐	☒	A statement on whether measurements were taken from distinct samples or whether the same sample was measured repeatedly
☐	☒	The statistical test(s) used AND whether they are one- or two-sided<br>*Only common tests should be described solely by name; describe more complex techniques in the Methods section.*
☐	☒	A description of all covariates tested
☐	☒	A description of any assumptions or corrections, such as tests of normality and adjustment for multiple comparisons
☐	☒	A full description of the statistical parameters including central tendency (e.g. means) or other basic estimates (e.g. regression coefficient) AND variation (e.g. standard deviation) or associated estimates of uncertainty (e.g. confidence intervals)
☐	☒	For null hypothesis testing, the test statistic (e.g. F, t, r) with confidence intervals, effect sizes, degrees of freedom and P value noted<br>*Give P values as exact values whenever suitable.*
☐	☒	For Bayesian analysis, information on the choice of priors and Markov chain Monte Carlo settings
☐	☒	For hierarchical and complex designs, identification of the appropriate level for tests and full reporting of outcomes
☐	☒	Estimates of effect sizes (e.g. Cohen's d, Pearson's r), indicating how they were calculated

Our web collection on statistics for biologists contains articles on many of the points above.

Software and code

Policy information about availability of computer code

Data collection	All healthy cell lines available from the HipSci Initiative were used
Data analysis	Analysis code is available at: https://github.com/andersonlab/macromap_eqtl. Additionally, the following programs/software were used:<br>QTLtools Version : 1.1<br>coloc v3.2-1<br>DESeq2_1.26.0<br>mashr_0.2.21<br>STAR_2.5.3a<br>bcftools Version: 1.10.2<br>samtools Version: 1.10 (using htslib 1.10)<br>FastQC v0.11.5<br>multiqc, version 1.3<br>featureCounts v1.5.3<br>CrossMap version 0.2.7<br>EIGENSTRAT 6.1.4

For manuscripts utilizing custom algorithms or software that are central to the research but not yet described in published literature, software must be made available to editors and reviewers. We strongly encourage code deposition in a community repository (e.g. GitHub). See the Nature Portfolio guidelines for submitting code & software for further information.

Data

Policy information about availability of data

All manuscripts must include a data availability statement. This statement should provide the following information, where applicable:

- Accession codes, unique identifiers, or web links for publicly available datasets
- A description of any restrictions on data availability
- For clinical datasets or third party data, please ensure that the statement adheres to our policy

Imputed genotype data for the HipSci lines are available from the European Nucleotide Archive (ENA) (PRJEB11749) and European Genome–phenome Archive (EGA) (EGAD00010000773). Unprocessed RNA-seq data are available from, EGA (EGAS00001002268). Full summary eQTL statistics, raw and processed counts and full colocalization results are available from Zenodo (https://doi.org/10.5281/zenodo.7967759).

Research involving human participants, their data, or biological material

Policy information about studies with human participants or human data. See also policy information about sex, gender (identity/presentation), and sexual orientation and race, ethnicity and racism.

Reporting on sex and gender	All information on sex for each cell line can be publicly queried at https://www.hipsci.org/#/lines. Sample identifiers in the raw and normalised counts files are identical to those listed in the HipSci browser. Sex information are also available in Supplementary Data 11.
Reporting on race, ethnicity, or other socially relevant groupings	All cell lines used in our study were obtained from individuals of European descent. Ethnicity and other population descriptors can be found for each cell line at https://www.hipsci.org/#/lines (e.g. https://www.hipsci.org/#/lines/HPSI0115i-aion_2). Ethnicity is also reported in Supplementary Data 11.
Population characteristics	All cell lines used in our study were obtained from individuals of European descent. Age group, sex and other population descriptors can be found for each cell line at https://www.hipsci.org/#/lines (e.g. https://www.hipsci.org/#/lines/HPSI0115i-aion_2). Also see Supplementary Data 11.
Recruitment	All healthy iPSC cell lines from European descent were obtained via the HipSci Initiative
Ethics oversight	HipSci samples were originally collected under ethical approval for induced pluripotent stem cell derivation (REC 09/H0304/77, V2 04/01/2013). Subsequent samples were collected following an updated consent (REC 09/H0304/77, V3 15/03/2013).

Note that full information on the approval of the study protocol must also be provided in the manuscript.

Field-specific reporting

Please select the one below that is the best fit for your research. If you are not sure, read the appropriate sections before making your selection.

☒ Life sciences ☐ Behavioural & social sciences ☐ Ecological, evolutionary & environmental sciences

For a reference copy of the document with all sections, see nature.com/documents/nr-reporting-summary-flat.pdf

Life sciences study design

All studies must disclose on these points even when the disclosure is negative.

Sample size	A total of 4,698 RNA-seq samples were analysed. This includes 177-202 RNA-seq samples per condition.
Data exclusions	Cell lines that were not successfully differentiated into macrophages (CD14+CD68+ purity assay < 90%) or were Mycoplasma infected were discarded
Replication	Experimental reproducibility:<br>Macrophage differentiation from 209 iPSC lines followed a standardized protocol with rigorous quality control at each stage. Only lines with >90% $CD14^+CD68^+$ purity were included. Cells were stimulated under 10 conditions at two timepoints, and RNA-seq libraries were randomized across 96-well plates to minimize batch effects. We used automated platforms for mRNA purification and library prep. Sample quality was verified using PCA, UMAP, and Match BAM to VCF (MBV), leading to correction or exclusion of mislabeled or contaminated samples.<br><br>Computational reproducibility:<br>All data analysis was done using scripted pipelines with version control, and software versions are listed in the Methods. Important factors like batch effects and genetic differences (genotype principal components) were included in the models as covariates. We shared all the code and summary statistics publicly on GitHub and Zenodo to ensure reproducibility.<br><br>Replication and validation:<br>As this is a computational study, formal replication through independent experimental validation was not performed. Reproducibility is ensured by the availability of all code and data for exact re-running of analyses. No evidence was found indicating irreproducible findings

	within the scope of this study.
Randomization	Samples were not randomized across experimental groups. All cell lines were derived from individuals with white European ancestry. Relevant experimental and demographic covariates were included in the differential splicing analyses (described in Methods). In sQTL analyses, a relevant number of principal components that miximise the number of splicing QTLs found were included as covariates (see Methods and Supplementary Figure 28).
Blinding	Blinding was not applied, as it was not relevant to this study. While we were aware of sample identities during data generation, RNA-seq libraries were randomized across 96-well plates to minimize batch effects. Data analysis was performed using scripted computational pipelines and standardized statistical tools (e.g., DESeq2, QTLtools, mashr), with no manual scoring or subjective interpretation. Group assignments (e.g., stimulation conditions, timepoints) were defined in metadata and used directly in the analysis. Sample swaps and mislabeling were identified and corrected, ensuring accurate group allocation throughout.

Reporting for specific materials, systems and methods

We require information from authors about some types of materials, experimental systems and methods used in many studies. Here, indicate whether each material, system or method listed is relevant to your study. If you are not sure if a list item applies to your research, read the appropriate section before selecting a response.

Materials & experimental systems

n/a	Involved in the study
☐	☒ Antibodies
☐	☒ Eukaryotic cell lines
☒	☐ Palaeontology and archaeology
☒	☐ Animals and other organisms
☒	☐ Clinical data
☒	☐ Dual use research of concern
☒	☐ Plants

Methods

n/a	Involved in the study
☒	☐ ChIP-seq
☒	☐ Flow cytometry
☒	☐ MRI-based neuroimaging

Antibodies

Antibodies used	Primary antibodies: Anti-CD14 (BioLegend, 301802) and anti-CD68 (Cell Signalling Technology 76437S)<br>Secondary antibodies: donkey anti-mouse AF647 and donkey anti-rabbit AF488
Validation	Anti-CD14 (BioLegend Cat# 301802, RRID:AB_314184) has verified reactivity in humans and is flow cyometry quality tested and CyTOF verified according to the manufacturer's product sheet. Recommended usage: flow cytometry staining (https://www.biolegend.com/en-us/products/purified-anti-human-cd14-antibody-797?pdf=true&displayInline=true&leftRightMargin=15&topBottomMargin=15&filename=Purified%20anti-human%20CD14%20Antibody.pdf&v=20250515124301).<br><br>Anti-CD68 (Cell Signaling Technology Cat# 76437, RRID:AB_2799882)According to the manufacturer's product page, the application key mentioned for anti-CD68 is " IHC-Immunohistochemistry IF-Immunofluorescence F-Flow Cytometry" with reported reactivity in humans. The manufacturer's website reports validation of the antibody for several applications including immunofluorescence imaging (https://www.cellsignal.com/products/primary-antibodies/cd68-d4b9c-xp-rabbit-mab/76437).

Eukaryotic cell lines

Policy information about cell lines and Sex and Gender in Research

Cell line source(s)	HipSci Initiative
Authentication	HipSci cell lines are authenticated via th European Collection of Authenticated Cell Cultures (ECCAC) and the European Bank for induced pluripotent Stem Cells (EBISC) (https://ebisc.org/).
Mycoplasma contamination	Cell lines were tested for mycoplasma contamination using PCR. Infected cell lines were discarded.
Commonly misidentified lines<br>(See ICLAC register)	None of our HipSci cell lines were reported in ICLAC v13

Plants

Seed stocks	*Report on the source of all seed stocks or other plant material used. If applicable, state the seed stock centre and catalogue number. If plant specimens were collected from the field, describe the collection location, date and sampling procedures.*
Novel plant genotypes	*Describe the methods by which all novel plant genotypes were produced. This includes those generated by transgenic approaches, gene editing, chemical/radiation-based mutagenesis and hybridization. For transgenic lines, describe the transformation method, the number of independent lines analyzed and the generation upon which experiments were performed. For gene-edited lines, describe the editor used, the endogenous sequence targeted for editing, the targeting guide RNA sequence (if applicable) and how the editor was applied.*
Authentication	*Describe any authentication procedures for each seed stock used or novel genotype generated. Describe any experiments used to assess the effect of a mutation and, where applicable, how potential secondary effects (e.g. second site T-DNA insertions, mosiacism, off-target gene editing) were examined.*
